# (Adipato-κ^2^ *O*,*O*′)di­aqua­[bis­(pyridin-2-yl-κ*N*)amine]­cobalt(II) trihydrate

**DOI:** 10.1107/S1600536813012981

**Published:** 2013-05-22

**Authors:** Zouaoui Setifi, Fatima Setifi, Graham Smith, Malika El-Ghozzi, Djamil-Azzeddine Rouag, Daniel Avignant, Hocine Merazig

**Affiliations:** a Département de Technologie, Faculté de Technologie, Université 20 Août 1955 de Skikda, 21000 Skikda, Algeria; b Unité de Recherche de Chimie de l’Environnement et Moléculaire Structurale (CHEMS), Université Constantine I, 25000 Constantine, Algeria; c Laboratoire de Chimie, Ingénierie Moléculaire et Nanostructures (LCIMN), Université Ferhat Abbas, Sétif I, 19000 Sétif, Algeria; d Science and Engineering Faculty, Queensland University of Technology, GPO Box 2434, Brisbane 4001, Australia; e Clermont Université, Université Blaise Pascal, Institut de Chimie de Clermont-Ferrand, BP 10448, 63000 Clermont-Ferrand, France; f CNRS UMR 6296, ICCF, BP 80026, 63171 Aubière, France

## Abstract

In the monomeric title complex, [Co(C_6_H_8_O_4_)(C_10_H_9_N_3_)(H_2_O)_2_]·3H_2_O, the distorted octa­hedral CoN_2_O_4_ coordination environment comprises two N-atom donors from the bidentate di­pyridyldi­amine ligand, two O-atom donors from one of the carboxyl­ate groups of the bidentate chelating adipate ligand and two water mol­ecules. In addition, there are three solvent water mol­ecules which are involved in both intra- and inter-unit O—H⋯O hydrogen-bonding inter­actions, which together with an amine–water N—H⋯O hydrogen bond produce a three-dimensional framework.

## Related literature

For the background to metal-di­carboxyl­ate complexes, see: Rao *et al.* (2004 ▶); Setifi *et al.* (2006 ▶, 2007 ▶); Wen *et al.* (2010 ▶).
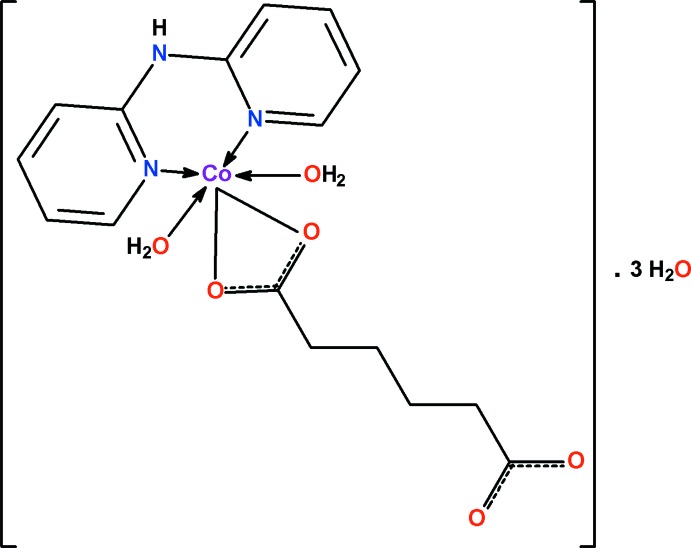


## Experimental

### 

#### Crystal data

[Co(C_6_H_8_O_4_)(C_10_H_9_N_3_)(H_2_O)_2_]·3H_2_O
*M* *_r_* = 464.34
Triclinic, 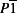

*a* = 9.9587 (3) Å
*b* = 10.5458 (3) Å
*c* = 11.0885 (3) Å
α = 100.887 (1)°
β = 105.891 (1)°
γ = 107.889 (1)°
*V* = 1017.38 (5) Å^3^
*Z* = 2
Mo *K*α radiation
μ = 0.90 mm^−1^
*T* = 296 K
0.26 × 0.21 × 0.18 mm

#### Data collection

Bruker APEXII CCD diffractometer
Absorption correction: multi-scan (*SADABS*; Bruker, 2008 ▶) *T*_min_ = 0.800, *T*_max_ = 0.855
28998 measured reflections
8197 independent reflections
6984 reflections with *I* > 2σ(*I*)
*R*_int_ = 0.024

#### Refinement

*R*[*F* ^2^ > 2σ(*F* ^2^)] = 0.030
*wR*(*F* ^2^) = 0.097
*S* = 0.99
8197 reflections
306 parameters
1 restraint
H atoms treated by a mixture of independent and constrained refinement
Δρ_max_ = 0.37 e Å^−3^
Δρ_min_ = −0.34 e Å^−3^

### 

Data collection: *APEX2* (Bruker, 2008 ▶); cell refinement: *SAINT* (Bruker, 2008 ▶); data reduction: *SAINT*; program(s) used to solve structure: *SHELXS97* (Sheldrick, 2008 ▶); program(s) used to refine structure: *SHELXL97* (Sheldrick, 2008 ▶); molecular graphics: *ORTEP-3 for Windows* (Farrugia, 2012 ▶) and *PLATON* (Spek, 2009 ▶); software used to prepare material for publication: *PLATON* and *publCIF* (Westrip, 2010 ▶).

## Supplementary Material

Crystal structure: contains datablock(s) global, I. DOI: 10.1107/S1600536813012981/sj5322sup1.cif

Structure factors: contains datablock(s) I. DOI: 10.1107/S1600536813012981/sj5322Isup2.hkl

Additional supplementary materials:  crystallographic information; 3D view; checkCIF report

## Figures and Tables

**Table 1:** Selected bond lengths (Å)

Co1—O1	2.0680 (10)
Co1—O2	2.3079 (9)
Co1—O5	2.0877 (12)
Co1—O6	2.1336 (9)
Co1—N1	2.0781 (9)
Co1—N2	2.0596 (10)

**Table 2:** Hydrogen-bond geometry (Å, °)

*D*—H⋯*A*	*D*—H	H⋯*A*	*D*⋯*A*	*D*—H⋯*A*
N3—H3⋯O9^i^	0.78 (2)	2.05 (2)	2.8228 (17)	172 (2)
O5—H51⋯O7^ii^	0.775 (19)	1.923 (19)	2.6933 (15)	172.7 (19)
O5—H52⋯O3^iii^	0.80 (2)	1.97 (2)	2.7706 (17)	173 (2)
O6—H61⋯O2^iv^	0.83 (2)	2.00 (2)	2.8278 (12)	178 (4)
O6—H62⋯O8	0.77 (2)	1.94 (2)	2.7056 (16)	170 (2)
O7—H71⋯O3^v^	0.74 (3)	2.56 (3)	3.2596 (18)	160 (3)
O7—H72⋯O1	0.89 (2)	1.86 (2)	2.7543 (16)	175 (2)
O8—H81⋯O4^v^	0.88 (3)	1.97 (3)	2.8221 (19)	163 (2)
O8—H82⋯O4^vi^	0.79 (3)	2.05 (3)	2.832 (2)	175 (3)
O9—H91⋯O3^vii^	0.77 (2)	2.12 (2)	2.8670 (17)	164 (2)
O9—H92⋯O3^v^	0.79 (3)	1.99 (3)	2.7452 (17)	160 (3)

Symmetry codes: (i) 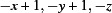; (ii) 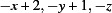; (iii) 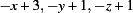; (iv) 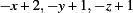; (v) 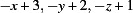; (vi) 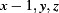; (vii) 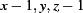.

**Fig. 1.**
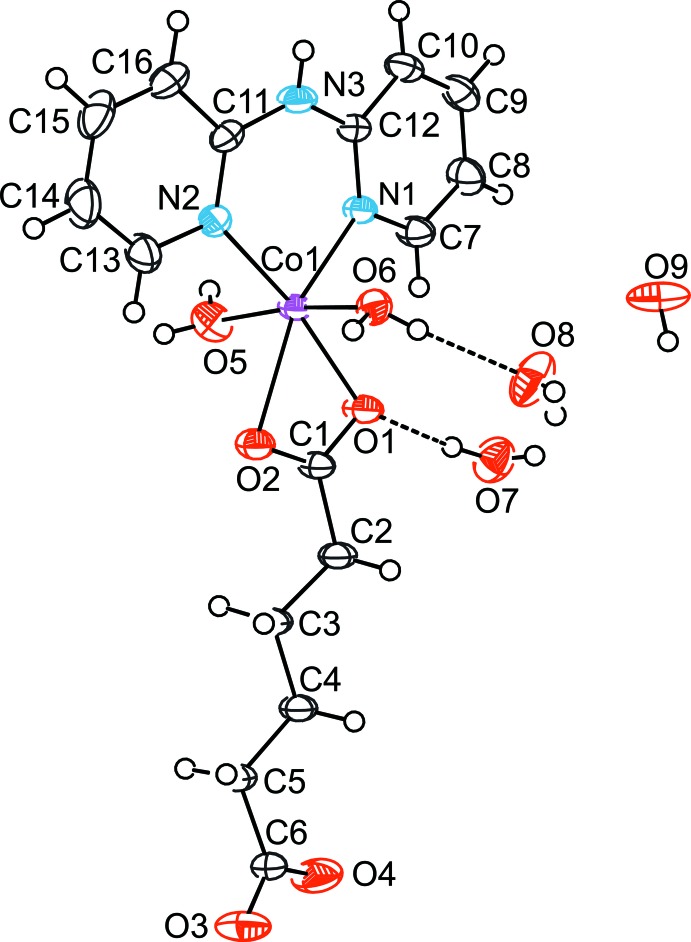
A view of the title compound with the atom numbering scheme. Displacement ellipsoids for non-H atoms are drawn at the 50% probability level.

**Fig. 2.**
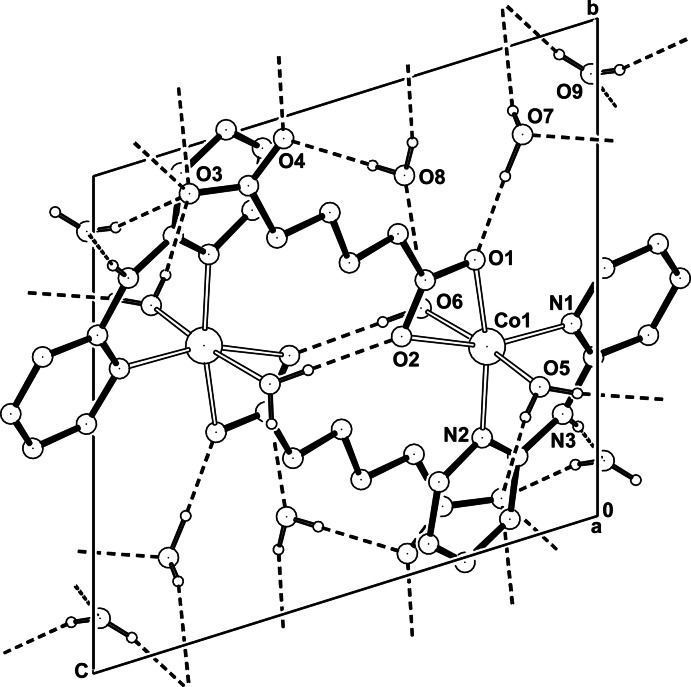
The crystal packing of the title compound in the unit cell viewed down a, showing hydrogen bonds as dashed lines. Non-associative H-atoms are omitted.

## supplementary crystallographic information

### Comment

Dicarboxylates have been widely used as ligands in metal coordination chemistry because they possess interesting features, such as: (i) the presence of two carboxylato groups capable of bidentate and monodentate linking modes, (ii) the possibility of obtaining mono- or dianionic forms, (iii) the probability of triply coordinated oxygen atoms and (iv) the possibility of forming secondary building blocks (Rao *et al.*, 2004). Their use as bridging ligands has generated metal-organic coordination polymers with diverse and interesting structural features (Setifi *et al.*, 2006, 2007; Wen *et al.*, 2010). Given the rich coordination chemistry and the flexibility of these anionic ligands, we are interested in using them in combination with other chelating co-ligands to explore their structural features and properties in the large field of molecular materials. This led us to the synthesis of the parent coordination compound, the title complex [Co(dpa)(adip)(H_2_O)_2_]. 3H_2_O, (where dpa is 2,2'-dipyridylamine and adip is the adipate dianion), and the structure is described herein.

In this monomeric complex, the distorted octahedral MN_2_O_4_ coordination sphere comprises two N-donors from the bidentate chelate dpa ligand, two carboxyl O-donors (O1, O2) from one of the carboxyl groups of the adipate ligand and two water molecules (O5, O6). In addition, there are three water molecules of solvation (O7–O9) (Fig. 1). The (N,*N*') interaction is essentially symmetric [Co—N, 2.0596 (10), 2.0781 (9) Å] (Table 1) but the (O,*O*') interaction is asymmetric [Co—O, 2.0680 (10), 2.3079 (9) Å], with a 'bite' angle of 59.68 (3)°. The second adipate carboxyl group is not involved in coordination.

In the crystal, both the coordinated water molecules and the solvent water molecules are involved in both intra- and inter-unit O—H···O hydrogen-bonding interactions. The amine N-atom of the dpa ligand is also hydrogen-bonded to a water molecule (O9) (Table 2), giving an overall three-dimensional framework structure (Fig. 2).

### Experimental

All reagents were purchased from commercial sources and used as received. Under aerobic conditions, an ethanolic solution of 2,2'-dipyridylamine (0.017 g, 5 ml) was added, with stirring at room temperature to an ethanolic solution of CoCl_2_.6H_2_O (0.024 g, 5 ml), resulting in a pink suspension. Adipic acid was dissolved in water (0.015 g, 10 ml) and added quickly to the mixture. The final solution was filtered and the filtrate allowed to evaporate in air for two weeks, giving brown crystals of the title compound suitable for X-ray diffraction analysis.

### Refinement

All H-atoms potentially involved in hydrogen-bonding were located from a difference-Fourier and both positional and isotropic displacement parameters were refined. Other H-atoms were placed in calculated positions with C—H(aromatic) = 0.93 Å or *C*—*H*(methylene) = 0.97 Å and with *U*_iso_(H) = 1.2*U*_eq_(C). Several reflections (8), considered to be affected by beam stop interference were omitted from the refinement.

### Crystal data

**Table:**

[Co(C_6_H_8_O_4_)(C_10_H_9_N_3_)(H_2_O)_2_]·3H_2_O	*Z* = 2
*M**_r_* = 464.34	*F*(000) = 486
Triclinic, *P*1	*D*_x_ = 1.516 Mg m^−^^3^
Hall symbol: -P 1	Mo *K*α radiation, λ = 0.71073 Å
*a* = 9.9587 (3) Å	Cell parameters from 9938 reflections
*b* = 10.5458 (3) Å	θ = 2.5–33.9°
*c* = 11.0885 (3) Å	µ = 0.90 mm^−^^1^
α = 100.887 (1)°	*T* = 296 K
β = 105.891 (1)°	Prism, brown
γ = 107.889 (1)°	0.26 × 0.21 × 0.18 mm
*V* = 1017.38 (5) Å^3^	

### Data collection

**Table:**

Bruker APEXII CCD diffractometer	8197 independent reflections
Radiation source: fine-focus sealed tube	6984 reflections with *I* > 2σ(*I*)
Graphite monochromator	*R*_int_ = 0.024
φ and ω scans	θ_max_ = 33.9°, θ_min_ = 3.3°
Absorption correction: multi-scan (*SADABS*; Bruker, 2008)	*h* = −15→15
*T*_min_ = 0.800, *T*_max_ = 0.855	*k* = −16→16
28998 measured reflections	*l* = −17→17

### Refinement

**Table:**

Refinement on *F*^2^	Primary atom site location: structure-invariant direct methods
Least-squares matrix: full	Secondary atom site location: difference Fourier map
*R*[*F*^2^ > 2σ(*F*^2^)] = 0.030	Hydrogen site location: inferred from neighbouring sites
*wR*(*F*^2^) = 0.097	H atoms treated by a mixture of independent and constrained refinement
*S* = 0.99	*w* = 1/[σ^2^(*F*_o_^2^) + (0.0677*P*)^2^ + 0.048*P*] where *P* = (*F*_o_^2^ + 2*F*_c_^2^)/3
8197 reflections	(Δ/σ)_max_ = 0.001
306 parameters	Δρ_max_ = 0.37 e Å^−^^3^
1 restraint	Δρ_min_ = −0.34 e Å^−^^3^

### Special details

**Table:**

Geometry. Bond distances, angles *etc*. have been calculated using the rounded fractional coordinates. All su's are estimated from the variances of the (full) variance-covariance matrix. The cell e.s.d.'s are taken into account in the estimation of distances, angles and torsion angles
Refinement. Refinement of *F*^2^ against ALL reflections. The weighted *R*-factor *wR* and goodness of fit *S* are based on *F*^2^, conventional *R*-factors *R* are based on *F*, with *F* set to zero for negative *F*^2^. The threshold expression of *F*^2^ > 2σ(*F*^2^) is used only for calculating *R*-factors(gt) *etc*. and is not relevant to the choice of reflections for refinement. *R*-factors based on *F*^2^ are statistically about twice as large as those based on *F*, and *R*- factors based on ALL data will be even larger.

### Fractional atomic coordinates and isotropic or equivalent isotropic displacement parameters (Å^2^)

**Table:**

	*x*	*y*	*z*	*U*_iso_*/*U*_eq_	
Co1	0.87882 (1)	0.40987 (1)	0.21923 (1)	0.0256 (1)	
O1	1.05309 (9)	0.59199 (9)	0.24329 (8)	0.0346 (2)	
O2	1.10712 (9)	0.48396 (9)	0.38874 (8)	0.0334 (2)	
O3	1.92943 (11)	0.90473 (12)	0.80972 (9)	0.0509 (3)	
O4	1.88720 (12)	0.95610 (12)	0.62266 (12)	0.0579 (3)	
O5	0.97049 (11)	0.29574 (11)	0.11448 (10)	0.0401 (3)	
O6	0.80952 (9)	0.52897 (9)	0.35009 (8)	0.0318 (2)	
N1	0.71591 (9)	0.40327 (9)	0.05172 (8)	0.0259 (2)	
N2	0.73045 (10)	0.22973 (9)	0.22883 (9)	0.0289 (2)	
N3	0.51381 (10)	0.22666 (11)	0.06928 (10)	0.0338 (2)	
C1	1.14696 (10)	0.58243 (11)	0.34175 (9)	0.0274 (2)	
C2	1.30419 (11)	0.69271 (12)	0.39566 (11)	0.0329 (3)	
C3	1.42382 (11)	0.66497 (12)	0.49212 (11)	0.0347 (3)	
C4	1.57881 (11)	0.77823 (11)	0.53102 (11)	0.0315 (3)	
C5	1.69945 (12)	0.76076 (12)	0.63591 (12)	0.0365 (3)	
C6	1.84988 (11)	0.88359 (11)	0.69272 (11)	0.0305 (2)	
C7	0.76159 (12)	0.49385 (12)	−0.01408 (11)	0.0332 (3)	
C8	0.66587 (14)	0.50906 (13)	−0.12028 (11)	0.0374 (3)	
C9	0.51360 (13)	0.42605 (14)	−0.16405 (10)	0.0367 (3)	
C10	0.46425 (12)	0.33147 (12)	−0.10088 (10)	0.0329 (3)	
C11	0.58275 (11)	0.17043 (10)	0.15844 (10)	0.0274 (2)	
C12	0.56943 (10)	0.32248 (10)	0.00812 (9)	0.0255 (2)	
C13	0.78977 (14)	0.16797 (14)	0.31479 (14)	0.0401 (3)	
C14	0.70567 (18)	0.05002 (16)	0.33525 (17)	0.0495 (5)	
C15	0.55197 (17)	−0.01009 (13)	0.26316 (16)	0.0469 (4)	
C16	0.48925 (14)	0.04862 (12)	0.17371 (13)	0.0387 (3)	
O7	1.14682 (14)	0.81433 (12)	0.14879 (11)	0.0492 (3)	
O8	0.88888 (16)	0.80611 (12)	0.38293 (15)	0.0613 (4)	
O9	0.80392 (11)	0.89324 (14)	0.01315 (13)	0.0604 (4)	
H2A	1.29990	0.77980	0.43830	0.0390*	
H2B	1.33620	0.70600	0.32230	0.0390*	
H3	0.426 (2)	0.1928 (19)	0.0392 (18)	0.050 (5)*	
H3A	1.42590	0.57550	0.45290	0.0420*	
H3B	1.39850	0.66010	0.57000	0.0420*	
H4A	1.60690	0.77760	0.45390	0.0380*	
H4B	1.57380	0.86830	0.56270	0.0380*	
H5A	1.71530	0.67790	0.59900	0.0440*	
H5B	1.66320	0.74550	0.70680	0.0440*	
H7	0.86380	0.54890	0.01450	0.0400*	
H8	0.70200	0.57340	−0.16210	0.0450*	
H9	0.44560	0.43450	−0.23560	0.0440*	
H10	0.36270	0.27410	−0.12970	0.0400*	
H13	0.89300	0.20800	0.36220	0.0480*	
H14	0.75020	0.01120	0.39570	0.0590*	
H15	0.49160	−0.08990	0.27540	0.0560*	
H16	0.38660	0.00850	0.12400	0.0460*	
H51	0.932 (2)	0.2581 (19)	0.0397 (19)	0.046 (5)*	
H52	1.002 (2)	0.242 (2)	0.143 (2)	0.065 (6)*	
H61	0.836 (3)	0.526 (3)	0.427 (2)	0.083 (7)*	
H62	0.838 (2)	0.607 (2)	0.3537 (19)	0.057 (5)*	
H71	1.117 (3)	0.868 (3)	0.168 (3)	0.107 (10)*	
H72	1.122 (2)	0.745 (2)	0.184 (2)	0.063 (6)*	
H81	0.958 (3)	0.868 (3)	0.367 (2)	0.075 (6)*	
H82	0.884 (3)	0.849 (3)	0.447 (3)	0.085 (8)*	
H91	0.833 (2)	0.880 (2)	−0.044 (2)	0.067 (6)*	
H92	0.870 (3)	0.949 (3)	0.077 (3)	0.085 (8)*	

### Atomic displacement parameters (Å^2^)

**Table:**

	*U*^11^	*U*^22^	*U*^33^	*U*^12^	*U*^13^	*U*^23^
Co1	0.0168 (1)	0.0288 (1)	0.0261 (1)	0.0034 (1)	0.0039 (1)	0.0117 (1)
O1	0.0216 (3)	0.0398 (4)	0.0325 (4)	0.0018 (3)	0.0020 (3)	0.0170 (3)
O2	0.0243 (3)	0.0370 (4)	0.0328 (3)	0.0047 (3)	0.0054 (3)	0.0156 (3)
O3	0.0306 (4)	0.0617 (6)	0.0328 (4)	−0.0007 (4)	−0.0034 (3)	0.0056 (4)
O4	0.0370 (5)	0.0520 (6)	0.0595 (6)	−0.0090 (4)	0.0017 (4)	0.0276 (5)
O5	0.0393 (5)	0.0504 (5)	0.0350 (4)	0.0211 (4)	0.0143 (4)	0.0141 (4)
O6	0.0328 (4)	0.0307 (4)	0.0303 (4)	0.0101 (3)	0.0098 (3)	0.0113 (3)
N1	0.0198 (3)	0.0288 (4)	0.0253 (3)	0.0054 (3)	0.0050 (3)	0.0103 (3)
N2	0.0245 (4)	0.0270 (4)	0.0352 (4)	0.0075 (3)	0.0106 (3)	0.0133 (3)
N3	0.0188 (4)	0.0360 (4)	0.0377 (4)	0.0013 (3)	0.0047 (3)	0.0144 (4)
C1	0.0182 (4)	0.0318 (4)	0.0265 (4)	0.0041 (3)	0.0052 (3)	0.0090 (3)
C2	0.0187 (4)	0.0328 (5)	0.0356 (5)	0.0015 (3)	0.0020 (3)	0.0100 (4)
C3	0.0201 (4)	0.0371 (5)	0.0347 (5)	0.0003 (4)	0.0015 (4)	0.0138 (4)
C4	0.0194 (4)	0.0324 (5)	0.0322 (4)	0.0017 (3)	0.0021 (3)	0.0109 (4)
C5	0.0228 (4)	0.0311 (5)	0.0409 (5)	0.0000 (4)	−0.0019 (4)	0.0148 (4)
C6	0.0198 (4)	0.0281 (4)	0.0342 (4)	0.0045 (3)	0.0035 (3)	0.0052 (4)
C7	0.0251 (4)	0.0388 (5)	0.0314 (4)	0.0058 (4)	0.0064 (4)	0.0171 (4)
C8	0.0370 (5)	0.0444 (6)	0.0326 (5)	0.0150 (5)	0.0096 (4)	0.0205 (4)
C9	0.0350 (5)	0.0473 (6)	0.0264 (4)	0.0204 (5)	0.0035 (4)	0.0111 (4)
C10	0.0223 (4)	0.0386 (5)	0.0288 (4)	0.0097 (4)	0.0010 (3)	0.0051 (4)
C11	0.0257 (4)	0.0227 (4)	0.0309 (4)	0.0045 (3)	0.0117 (3)	0.0068 (3)
C12	0.0198 (4)	0.0265 (4)	0.0247 (4)	0.0059 (3)	0.0046 (3)	0.0050 (3)
C13	0.0338 (5)	0.0419 (6)	0.0522 (7)	0.0166 (5)	0.0152 (5)	0.0269 (5)
C14	0.0541 (8)	0.0447 (7)	0.0679 (9)	0.0250 (6)	0.0285 (7)	0.0362 (7)
C15	0.0548 (8)	0.0299 (5)	0.0661 (9)	0.0130 (5)	0.0331 (7)	0.0243 (6)
C16	0.0340 (5)	0.0280 (5)	0.0469 (6)	0.0008 (4)	0.0168 (5)	0.0103 (4)
O7	0.0603 (7)	0.0403 (5)	0.0440 (5)	0.0140 (5)	0.0175 (5)	0.0159 (4)
O8	0.0727 (8)	0.0345 (5)	0.0813 (9)	0.0112 (5)	0.0427 (7)	0.0201 (5)
O9	0.0245 (4)	0.0761 (8)	0.0492 (6)	−0.0076 (5)	0.0069 (4)	0.0033 (5)

### Geometric parameters (Å, º)

**Table:**

Co1—O1	2.0680 (10)	C2—C3	1.5111 (17)
Co1—O2	2.3079 (9)	C3—C4	1.5177 (17)
Co1—O5	2.0877 (12)	C4—C5	1.5106 (17)
Co1—O6	2.1336 (9)	C5—C6	1.5172 (17)
Co1—N1	2.0781 (9)	C7—C8	1.3681 (17)
Co1—N2	2.0596 (10)	C8—C9	1.387 (2)
O1—C1	1.2715 (13)	C9—C10	1.3705 (18)
O2—C1	1.2537 (14)	C10—C12	1.4064 (15)
O3—C6	1.2569 (15)	C11—C16	1.4070 (17)
O4—C6	1.2387 (17)	C13—C14	1.368 (2)
O5—H51	0.775 (19)	C14—C15	1.388 (3)
O5—H52	0.80 (2)	C15—C16	1.374 (2)
O6—H62	0.77 (2)	C2—H2A	0.9700
O6—H61	0.83 (2)	C2—H2B	0.9700
O7—H71	0.74 (3)	C3—H3A	0.9700
O7—H72	0.89 (2)	C3—H3B	0.9700
O8—H81	0.88 (3)	C4—H4B	0.9700
O8—H82	0.79 (3)	C4—H4A	0.9700
O9—H92	0.79 (3)	C5—H5A	0.9700
O9—H91	0.77 (2)	C5—H5B	0.9700
N1—C12	1.3350 (14)	C7—H7	0.9300
N1—C7	1.3527 (15)	C8—H8	0.9300
N2—C11	1.3363 (15)	C9—H9	0.9300
N2—C13	1.3580 (17)	C10—H10	0.9300
N3—C12	1.3815 (15)	C13—H13	0.9300
N3—C11	1.3785 (15)	C14—H14	0.9300
N3—H3	0.78 (2)	C15—H15	0.9300
C1—C2	1.5059 (16)	C16—H16	0.9300
			
O1—Co1—O2	59.68 (3)	C9—C10—C12	119.02 (11)
O1—Co1—O5	89.56 (4)	N3—C11—C16	116.66 (11)
O1—Co1—O6	88.57 (4)	N2—C11—N3	121.87 (10)
O1—Co1—N1	100.35 (4)	N2—C11—C16	121.46 (10)
O1—Co1—N2	169.33 (4)	N1—C12—N3	121.29 (9)
O2—Co1—O5	84.94 (4)	N3—C12—C10	116.80 (10)
O2—Co1—O6	87.44 (3)	N1—C12—C10	121.92 (10)
O2—Co1—N1	159.98 (4)	N2—C13—C14	123.44 (14)
O2—Co1—N2	109.79 (4)	C13—C14—C15	118.04 (15)
O5—Co1—O6	172.04 (4)	C14—C15—C16	119.82 (14)
O5—Co1—N1	94.09 (4)	C11—C16—C15	118.94 (13)
O5—Co1—N2	91.18 (4)	C1—C2—H2B	108.00
O6—Co1—N1	93.86 (4)	H2A—C2—H2B	107.00
O6—Co1—N2	89.24 (4)	C3—C2—H2A	108.00
N1—Co1—N2	90.21 (4)	C3—C2—H2B	108.00
Co1—O1—C1	95.33 (7)	C1—C2—H2A	108.00
Co1—O2—C1	84.88 (6)	C4—C3—H3B	109.00
Co1—O5—H51	122.6 (16)	H3A—C3—H3B	108.00
Co1—O5—H52	120.3 (15)	C4—C3—H3A	109.00
H51—O5—H52	104 (2)	C2—C3—H3A	109.00
Co1—O6—H61	116 (2)	C2—C3—H3B	109.00
Co1—O6—H62	112.1 (15)	C3—C4—H4A	109.00
H61—O6—H62	107 (3)	C3—C4—H4B	109.00
H71—O7—H72	112 (3)	C5—C4—H4A	109.00
H81—O8—H82	103 (3)	C5—C4—H4B	109.00
H91—O9—H92	111 (3)	H4A—C4—H4B	108.00
C7—N1—C12	117.69 (9)	C6—C5—H5A	109.00
Co1—N1—C12	125.38 (7)	H5A—C5—H5B	108.00
Co1—N1—C7	116.84 (8)	C4—C5—H5B	109.00
Co1—N2—C13	116.29 (9)	C6—C5—H5B	109.00
C11—N2—C13	118.28 (10)	C4—C5—H5A	109.00
Co1—N2—C11	125.44 (7)	N1—C7—H7	118.00
C11—N3—C12	132.59 (11)	C8—C7—H7	118.00
C12—N3—H3	110.7 (14)	C9—C8—H8	121.00
C11—N3—H3	116.2 (14)	C7—C8—H8	121.00
O1—C1—C2	116.83 (10)	C10—C9—H9	120.00
O2—C1—C2	123.13 (10)	C8—C9—H9	120.00
O1—C1—O2	120.04 (10)	C9—C10—H10	120.00
C1—C2—C3	116.75 (10)	C12—C10—H10	121.00
C2—C3—C4	111.55 (10)	N2—C13—H13	118.00
C3—C4—C5	113.16 (10)	C14—C13—H13	118.00
C4—C5—C6	114.59 (10)	C15—C14—H14	121.00
O4—C6—C5	119.03 (11)	C13—C14—H14	121.00
O3—C6—O4	124.09 (12)	C14—C15—H15	120.00
O3—C6—C5	116.88 (11)	C16—C15—H15	120.00
N1—C7—C8	123.70 (12)	C11—C16—H16	120.00
C7—C8—C9	118.30 (12)	C15—C16—H16	121.00
C8—C9—C10	119.36 (11)		
			
O2—Co1—O1—C1	1.54 (6)	C12—N1—C7—C8	1.49 (17)
O5—Co1—O1—C1	−82.85 (7)	Co1—N1—C12—N3	−4.11 (15)
O6—Co1—O1—C1	89.41 (7)	Co1—N1—C12—C10	175.23 (8)
N1—Co1—O1—C1	−176.92 (7)	C7—N1—C12—N3	179.59 (10)
O1—Co1—O2—C1	−1.56 (6)	C7—N1—C12—C10	−1.07 (16)
O5—Co1—O2—C1	90.90 (7)	Co1—N2—C11—N3	0.16 (15)
O6—Co1—O2—C1	−91.40 (7)	Co1—N2—C11—C16	−178.58 (9)
N1—Co1—O2—C1	2.85 (14)	C13—N2—C11—N3	179.90 (11)
N2—Co1—O2—C1	−179.67 (7)	C13—N2—C11—C16	1.16 (17)
O1—Co1—N1—C7	11.20 (9)	Co1—N2—C13—C14	178.23 (13)
O1—Co1—N1—C12	−165.14 (9)	C11—N2—C13—C14	−1.5 (2)
O2—Co1—N1—C7	7.33 (16)	C12—N3—C11—N2	17.69 (19)
O2—Co1—N1—C12	−169.01 (9)	C12—N3—C11—C16	−163.51 (12)
O5—Co1—N1—C7	−79.11 (9)	C11—N3—C12—N1	−15.42 (19)
O5—Co1—N1—C12	104.56 (9)	C11—N3—C12—C10	165.21 (12)
O6—Co1—N1—C7	100.44 (9)	O1—C1—C2—C3	−167.09 (10)
O6—Co1—N1—C12	−75.89 (9)	O2—C1—C2—C3	12.39 (16)
N2—Co1—N1—C7	−170.31 (9)	C1—C2—C3—C4	175.72 (9)
N2—Co1—N1—C12	13.36 (9)	C2—C3—C4—C5	175.19 (10)
O2—Co1—N2—C11	169.49 (9)	C3—C4—C5—C6	−170.72 (10)
O2—Co1—N2—C13	−10.25 (10)	C4—C5—C6—O3	150.64 (12)
O5—Co1—N2—C11	−105.46 (10)	C4—C5—C6—O4	−29.52 (17)
O5—Co1—N2—C13	74.80 (10)	N1—C7—C8—C9	−0.72 (19)
O6—Co1—N2—C11	82.49 (9)	C7—C8—C9—C10	−0.50 (19)
O6—Co1—N2—C13	−97.25 (9)	C8—C9—C10—C12	0.87 (18)
N1—Co1—N2—C11	−11.37 (9)	C9—C10—C12—N1	−0.08 (16)
N1—Co1—N2—C13	168.89 (9)	C9—C10—C12—N3	179.29 (11)
Co1—O1—C1—O2	−2.83 (11)	N2—C11—C16—C15	0.00 (19)
Co1—O1—C1—C2	176.67 (8)	N3—C11—C16—C15	−178.80 (13)
Co1—O2—C1—O1	2.54 (10)	N2—C13—C14—C15	0.7 (2)
Co1—O2—C1—C2	−176.93 (10)	C13—C14—C15—C16	0.5 (2)
Co1—N1—C7—C8	−175.13 (10)	C14—C15—C16—C11	−0.9 (2)

### Hydrogen-bond geometry (Å, º)

**Table:**

*D*—H···*A*	*D*—H	H···*A*	*D*···*A*	*D*—H···*A*
N3—H3···O9^i^	0.78 (2)	2.05 (2)	2.8228 (17)	172 (2)
O5—H51···O7^ii^	0.775 (19)	1.923 (19)	2.6933 (15)	172.7 (19)
O5—H52···O3^iii^	0.80 (2)	1.97 (2)	2.7706 (17)	173 (2)
O6—H61···O2^iv^	0.83 (2)	2.00 (2)	2.8278 (12)	178 (4)
O6—H62···O8	0.77 (2)	1.94 (2)	2.7056 (16)	170 (2)
O7—H71···O3^v^	0.74 (3)	2.56 (3)	3.2596 (18)	160 (3)
O7—H72···O1	0.89 (2)	1.86 (2)	2.7543 (16)	175 (2)
O8—H81···O4^v^	0.88 (3)	1.97 (3)	2.8221 (19)	163 (2)
O8—H82···O4^vi^	0.79 (3)	2.05 (3)	2.832 (2)	175 (3)
O9—H91···O3^vii^	0.77 (2)	2.12 (2)	2.8670 (17)	164 (2)
O9—H92···O3^v^	0.79 (3)	1.99 (3)	2.7452 (17)	160 (3)

Symmetry codes: (i) −*x*+1, −*y*+1, −*z*; (ii) −*x*+2, −*y*+1, −*z*; (iii) −*x*+3, −*y*+1, −*z*+1; (iv) −*x*+2, −*y*+1, −*z*+1; (v) −*x*+3, −*y*+2, −*z*+1; (vi) *x*−1, *y*, *z*; (vii) *x*−1, *y*, *z*−1.
